# An essential contractile ring protein controls cell division in *Plasmodium falciparum*

**DOI:** 10.1038/s41467-019-10214-z

**Published:** 2019-05-16

**Authors:** Rachel M. Rudlaff, Stephan Kraemer, Vincent A. Streva, Jeffrey D. Dvorin

**Affiliations:** 1 Biological and Biomedical Sciences, Harvard Medical School, Boston, MA 02115 USA; 2 Division of Infectious Diseases, Boston Children’s Hospital, Boston, MA 02115 USA; 3 Center for Nanoscale Systems, Harvard University, Boston, MA 02138 USA; 4 Department of Pediatrics, Harvard Medical School, Boston, MA 02115 USA

**Keywords:** Scanning electron microscopy, Transmission electron microscopy, Cell division, Parasite biology

## Abstract

During the blood stage of human malaria, *Plasmodium falciparum* parasites divide by schizogony—a process wherein components for several daughter cells are produced within a common cytoplasm and then segmentation, a synchronized cytokinesis, produces individual invasive daughters. The basal complex is hypothesized to be required for segmentation, acting as a contractile ring to establish daughter cell boundaries. Here we identify an essential component of the basal complex which we name PfCINCH. Using three-dimensional reconstructions of parasites at electron microscopy resolution, we show that while parasite organelles form and divide normally, PfCINCH-deficient parasites develop inviable conjoined daughters that contain components for multiple cells. Through biochemical evaluation of the PfCINCH-containing complex, we discover multiple previously undescribed basal complex proteins. Therefore, this work provides genetic evidence that the basal complex is required for precise segmentation and lays the groundwork for a mechanistic understanding of how the parasite contractile ring drives cell division.

## Abstract (web summary)

Schizogony is essential for blood stage infection of *Plasmodium* parasites and produces several daughter cells. Here, Rudlaff et al. identify PfCINCH and interacting proteins as essential components of the basal complex required to establish daughter cell boundaries.

## Introduction

Malaria remains a global threat to human health with 200 million cases and 400,000 resulting deaths in 2016^1^. Malaria is caused by multiple species of *Plasmodium* parasites, the deadliest of which is *Plasmodium falciparum*. Despite the importance of this pathogen, many of the biological mechanisms that underlie critical parasite processes remain elusive. This knowledge gap is largely due to the divergent nature of *Plasmodium* parasites—approximately 50% of *Plasmodium* genes have no homology to genes of known function, and even in relatively conserved processes, *Plasmodium* biology differs greatly from that of model organisms^2,3^. In particular, in the clinically important blood stage of infection, the parasite undergoes segmentation, rather than fission, to produce daughter cells^4,5^.

In the intraerythrocytic life cycle, the parasite grows and divides inside human red blood cells (RBCs)^6–8^. This cycle begins when an invasive merozoite form of the parasite enters a RBC. Following invasion, the parasite replicates its genetic material and organelles over about 44 h to produce components for ~20–32 daughter cells within the common cytoplasm of a schizont. In the final hours of the cycle, daughter merozoites are formed by segmentation, wherein organelles and nuclei are partitioned and packaged to produce individual merozoites^9^. The asexual life cycle culminates after 48 h with egress, the process by which merozoites are rapidly released from the RBC to reinitiate the cycle^10–13^.

**Fig. 1:**
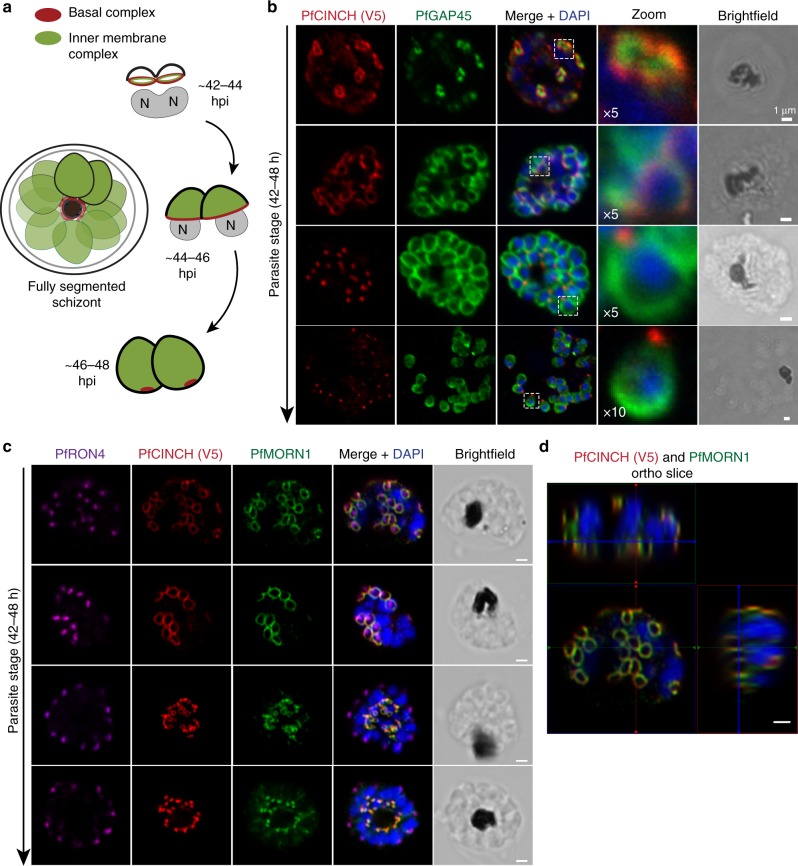
PfCINCH (*P. falciparum* *c*oord*i*nator of *n*ascent *c*ell detac*h*ment) is a member of the *Plasmodium* basal complex. **a** Schematic of inner membrane complex (IMC) and basal complex formation in schizogony. At 42–44 h post invasion, basal complex formation is nucleated near the apical ends of forming daughter cells at the basal end of the IMC. As segmentation progresses from ~44–46 h, the basal complex resides at the leading edge of membrane formation, while the IMC stretches from the basal complex to the apical pole of the cell. When segmentation is complete at ~46–48 h, the IMC extends from the apical to the basal pole of the cell, while the basal complex resides exclusively at the posterior end of the merozoite. **b** Confocal microscopy of PfCINCH and PfGAP45 (IMC marker) throughout segmentation. **c** Airyscan super-resolution microscopy (1.7-fold resolution increase) of PfCINCH and PfMORN1 (basal complex marker) throughout segmentation. **d** Ortho-slice of PfMORN1 and PfCINCH at the beginning of segmentation. All scale bars 1 µm

The inner membrane complex (IMC) is a divergent organelle involved in the coordination of segmentation^4,14^. It is a double lipid bilayer formed from flattened vesicles that, together with associated proteins, lies interior to the PPM^15,16^ and is hypothesized to define the shape of, provide structural rigidity to, and be involved in cell division of nascent daughter parasites^16–19^. It additionally anchors many of the glideosome proteins required for actinomyosin-based gliding motility^20–26^. Beneath the IMC lies a network of alveolins—intermediate filament-like proteins that provide support to the IMC^27–38^. The IMC forms during segmentation, during which it envelopes the daughter cell and forms a cylinder-like structure around its contents that extends from the merozoite’s apical to basal end, excluding its poles. At the apical end of the merozoite, the apical ring is hypothesized to nucleate the formation of sub-pellicular microtubules that stabilize the IMC on its cytoplasmic face^39–41^. The basal complex resides at the posterior end of the forming IMC, and exclusively at the basal pole in mature merozoites (Fig. 1a). This complex is hypothesized to act as a contractile ring to constrain and define the nascent cell’s boundaries, to generate force to pull the IMC down the length of the daughter cell, and to release newly formed merozoites by completing membrane fusion at their basal ends^34,42–46^.

Multiple basal complex proteins have been identified in the related apicomplexan parasite *Toxoplasma gondii*, including TgMORN1^47^, TgHAD2a^48^, TgDLC^49^, TgCentrin2^42^, TgIMC5, TgIMC8, TgIMC9, TgIMC13, TgIMC15^34^, Tg14-3-3, TgMSC1a^50^, TgMyoC^51^, and TgMyoJ^52^. Of these, TgMORN1, TgMyoJ, and TgHAD2a have been shown to be important for *T. gondii* replication. Comparatively less is known about the basal complex in *P. falciparum*. Only three components are known—PfMORN1^46^, PfHAD2^48^, and the *Plasmodium-*specific PfBTP1^45^—and none has been functionally evaluated. While there is some overlap between *T. gondii* and *P. falciparum* basal complex proteins (e.g., TgMORN1/PfMORN1 and TgHAD2a/PfHAD2), the *T. gondii* basal complex has been investigated primarily during the tachyzoite stage of this parasite’s life cycle, wherein each nuclear division is immediately followed by cytokinesis and formation of two daughter parasites through the process of endodyogeny^4^. In contrast, in *Plasmodium* blood stage replication, multiple cycles of nuclear division precede segmentation during which >12 daughter parasites are formed simultaneously. Because of the striking difference in morphology, as well as the >250 million years of evolution separating these parasites^53^, *P. falciparum* daughter cell assembly may be profoundly different, and the extent to which *Plasmodium* proteins mirror the functions of *Toxoplasma* basal complex proteins is unknown. Evidence to support the hypothesis that the *P. falciparum* basal complex is essential for asexual replication has thus far not been generated, and it is unknown whether this process is conserved or even required in *Plasmodium*.

In the current study, we identify an essential conserved *Plasmodium*-specific protein (PF3D7_0407800) that localizes to the basal complex and is required for daughter cell segmentation, which we name *P. falciparum* *c*oord*i*nator of *n*ascent *c*ell detac*h*ment, or PfCINCH. We provide the first direct evidence for an essential component of the *Plasmodium* basal complex in the asexual stage of the *Plasmodium* life cycle, demonstrate that proper segmentation is not a prerequisite for egress, and identify multiple new components of the *P. falciparum* basal complex for future investigation.

## Results

### PfCINCH is a member of the *Plasmodium* basal complex

In ongoing studies to characterize novel *P. falciparum* proteins, we identified PF3D7_0407800, a conserved *Plasmodium* protein of unknown function, which we named PfCINCH. To interrogate its localization, we fused the spaghetti monster V5 (smV5) epitope tag^54,55^ to the carboxy terminus of PfCINCH. The resulting transgenic parasite strain was named PfCINCH^smV5-Tet^ (Supplementary Fig. 1a and b). This line was generated in a 3D7 background parasite strain where PfCDPK5 is fused in a marker-free fashion to nanoluciferase and three copies of the hemagglutinin epitope tag. This parasite strain (denoted parental) has no detectable differential development or growth compared to wild-type 3D7 parasites (Supplementary Fig. 1c). We observed that PfCINCH forms small rings at the apical end of the nascent daughter cell prior to the final synchronous round of cell division (Fig. 1b). As segmentation progresses, PfCINCH tracks along the outer rim of the daughter cell, at the leading edge of the IMC, defined by positive staining for PfGAP45. Finally, when segmentation is complete, PfCINCH resides exclusively at the basal end of merozoites (Fig. 1b). This expression pattern mirrors that of the known *Plasmodium* basal complex proteins, PfBTP1 and PfMORN1^45,46^. To confirm that PfCINCH localizes to the basal complex, we co-stained with antibodies directed against PfCINCH and PfMORN1. They overlap at all stages of segmentation, indicating that PfCINCH is a member of the basal complex (Fig. 1c, d).

### PfCINCH is required for asexual stage replication

**Fig. 2:**
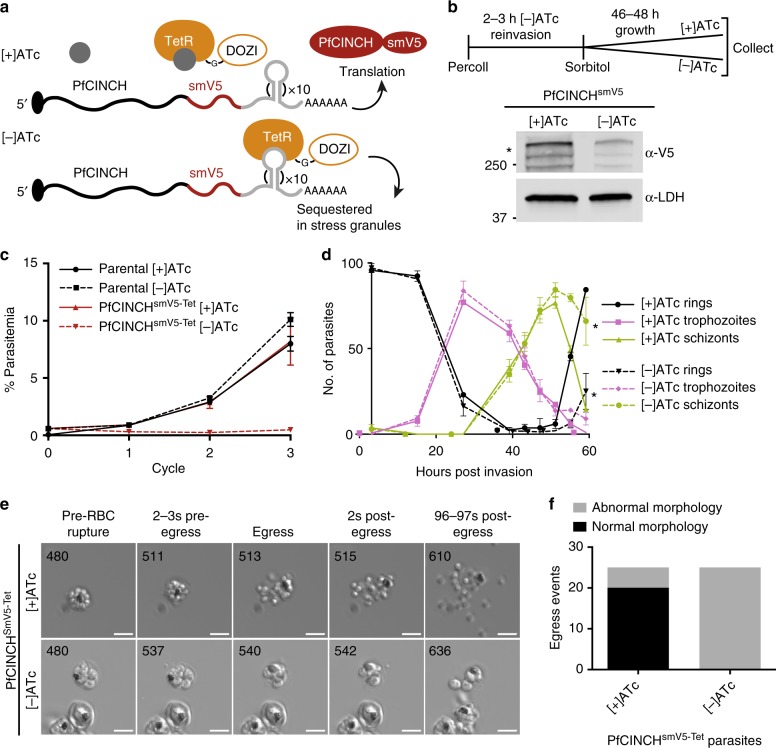
PfCINCH (*P. falciparum* *c*oord*i*nator of *n*ascent *c*ell detac*h*ment) is required for asexual stage replication. **a** Schematic of the TetR-DOZI system for translational repression. **b** Schematic of anhydrotetracycline (ATc) washout procedure and immunoblot of PfCINCH protein levels [+] and [−]ATc. Asterisk corresponds to the primary translation product of PfCINCH^smV5^. **c** Replication curve of PfCINCH^smV5-Tet^ and parental parasites over three replication cycles [+] and [−]ATc. Mean ± SD for triplicate wells shown. **d** Staging curve of PfCINCH^smV5-Tet^ parasites [+]/[−]ATc over one replication cycle. Mean ± SD for triplicate wells shown. **e** Time-lapse differential interference contrast (DIC) micrographs of PfCINCH^smV5-Tet^ parasites [+]/[−]ATc at egress. Scale bars 5 µm. **f** Graph of normal and abnormal egress events observed from PfCINCH^smV5-Tet^ parasites [+]/[−]ATc. smV5, spaghetti monster V5; TetR, Tet-repressor. Source data are provided as a Source Data file

To determine the function of PfCINCH during asexual parasite development, we applied the Tet repressor (TetR)-binding aptamer system^56,57^ to conditionally repress PfCINCH translation. In the PfCINCH^smV5-Tet^ parasite strain described above, we included ten copies of the TetR-binding aptamer at the 3′ end and expressed a TetR-DOZI fusion protein using the 2A skip peptide within the resistance cassette^58^. In the absence of the TetR ligand, anhydrotetracycline (ATc), TetR binds the aptamer array and the PfDOZI portion of the protein sequesters the targeted messenger RNA (mRNA) in parasite stress granules, preventing translation^56^ (Fig. 2a).

PfCINCH^smV5-Tet^ parasites maintained on ATc replicate at a similar rate to the parental line. To examine the efficiency of PfCINCH^smV5-Tet^ knockdown, we collected parasites maintained [+]/[−]ATc 48 h after washout. By quantitative immunoblot, we observe a 69 ± 11% knockdown of PfCINCH^smV5^ protein (Fig. 2b). Importantly, at this level of knockdown, we observe a complete replication block within the first cycle of growth (Fig. 2c). When ATc concentrations are titrated from 500 to 0 nM, we observe intermediate growth (Supplementary Fig. 2). These findings were confirmed in a PfCINCH^smV5-Tet^ line generated in a wild-type 3D7 background with no genetic modifications (Supplementary Fig. 3).

We hypothesized that the replication defect in PfCINCH-deficient parasites was due to a failure to complete segmentation. To test this hypothesis, we monitored development and growth of highly synchronized parasites by light microscopy. Parasites mature normally through the ring (0–24 h post invasion [hpi]) and trophozoite stages (24–36 hpi) and through early schizogony (36–42 hpi). The parasitemia and developmental stage remain similar between the [+]ATc and [−]ATc parasites throughout the first asexual cycle (Fig. 2d). We observed no significant difference in the number of genomes present in mature schizonts, determined by the mean fluorescence intensity (MFI) of parasites stained with the DNA-specific dye, SYBR Green I, under [+]ATc and [−]ATc conditions (Supplementary Fig. 4a and b). At 56 hpi, the schizont parasitemia drops equivalently in the [+]ATc and [−]ATc parasites (Supplementary Fig. 4c). Because of the decreased ring parasitemia, the proportion of total remaining parasites that are schizonts is relatively higher in the [−]ATc condition. These results suggest that PfCINCH-deficient parasites can egress and release merozoites, but these mutant daughter cells do not reinvade.

To test the hypothesis that PfCINCH-deficient parasites undergo physiologic egress, we performed time-lapse microscopy on parasites treated with compound 1 (4-[2-(4-fluorophenyl)-5-(1-methylpiperidine-4-yl)-1*H*-pyrrol-3-yl]pyridine or C1), a reversible protein kinase G (PfPKG) inhibitor^59,60^. C1 treatment arrests schizont maturation prior to egress, and when washed out allows maturation to proceed through egress^61^. We added C1 to 44–46 hpi schizonts for 6 h, either washed cells with C1-free or C1-containing media, and used time-lapse microscopy to visualize cells from 10 to 25 min after washout. As expected, parasites maintained on C1 do not egress. When C1 is washed out in both conditions, parasites egress rapidly (Supplementary Movies 1 and 2). Interestingly, PfCINCH-deficient schizonts release morphologically aberrant merozoites that by  time-lapse microscopy appear to vary in size and frequently remain attached to each other or the digestive vacuole (Fig. 2e). To assess the morphology of PfCINCH-deficient parasites at egress, we monitored 25 individual egress events in both [+]ATc and [−]ATc parasites without C1 treatment. We scored the egress event as aberrant if >3 daughter cells remained attached to the residual body or to one another. In [+]ATc parasites 80% of the egress events release fully segmented merozoites, but in five cases >3 daughter cells remained attached to the residual body. We hypothesize that the presence of the Tet aptamer array may cause a minor decrease in protein expression compared to a wild-type parasite (i.e., hypomorph), and therefore, explain the observed defect in [+]ATc parasites. In contrast, all 25 monitored PfCINCH-deficient schizonts released aberrant daughter cells that remained attached to each other (Fig. 2f). Together, these results show that, even when parasites have undergone improper cytokinesis, the process by which parasites trigger the egress signaling cascade remains intact.

### Segmentation is impaired in PfCINCH-deficient parasites

**Fig. 3:**
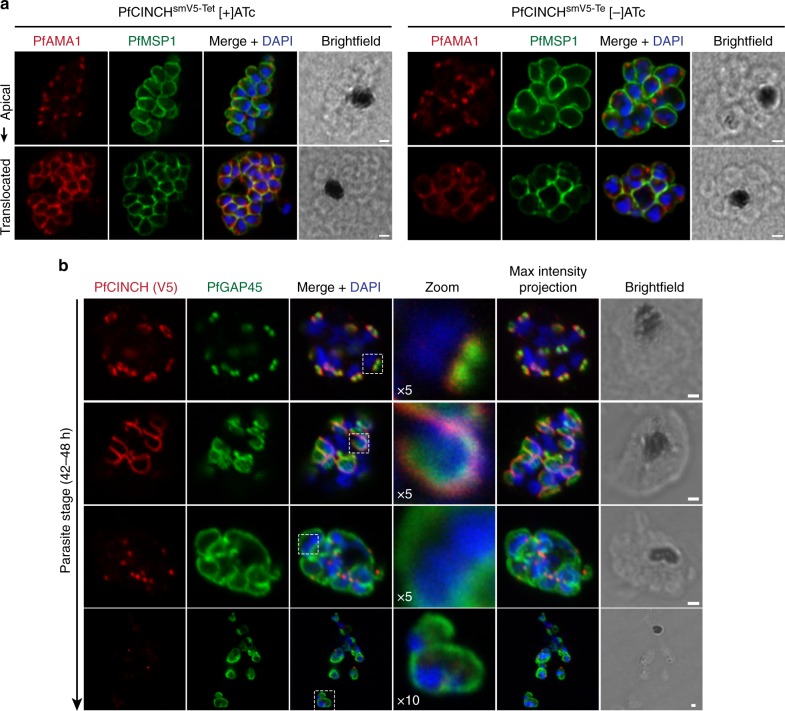
Segmentation is impaired in PfCINCH (*P. falciparum* *c*oord*i*nator of *n*ascent *c*ell detac*h*ment)-deficient parasites. **a** Airyscan super-resolution micrographs of merozoite plasma membrane and micronemes, stained with antibodies against *P. falciparum* merozoite surface protein 1 (PfMSP1) and PfAMA1, respectively, in E64-treated schizonts cultivated with and without anhydrotetracycline (ATc). Following the egress trigger, *P. falciparum* apical membrane antigen 1 (PfAMA1) is translocated from micronemes to the merozoite membrane (bottom panels). **b** Confocal micrographs of PfGAP45 (IMC marker) and residual PfCINCH over the course of segmentation in PfCINCH-deficient parasites. PfCINCH is visible in schizonts, but forms relatively larger rings that surround multiple nuclei as merozoites are formed. All scale bars 1 µm

We examined formation of the parasite plasma membrane (PPM) using an antibody against merozoite surface protein 1 (PfMSP1), which coats the PPM of newly formed daughter cells^62^. PfMSP1 staining revealed that, consistent with our model, PPM surrounds agglomerates of daughter cells in PfCINCH-deficient schizonts (Fig. 3a). PfCINCH^smV5-Tet^ parasites maintained on ATc have normal morphology and PfMSP1 stains membrane that surrounds individual nuclei. *Plasmodium falciparum* belongs to the Apicomplexan phylum of parasites that are distinguished by the presence of specialized apical secretory organelles, known as rhoptries, micronemes, and dense granules. We simultaneously examined microneme formation and discharge with a primary antibody against PfAMA1 (*P. falciparum* apical membrane antigen 1), a microneme-resident protein^63^. Micronemes are present in PfCINCH-deficient parasites and are even discharged upon the egress trigger to the PPM, encircling the poorly segmented daughter cell agglomerates (Fig. 3a). To examine IMC formation, we evaluated PfGAP45^23^, a glideosome-associated protein that connects the PPM and IMC, over the course of schizogony. We co-stained with an antibody directed against the V5 epitope to examine residual PfCINCH^smV5^ in knockdown parasites. In parasites maintained on ATc, the IMC initially forms as two small rings at the apical end of the daughters and progresses along the outer edge of the nascent cell. Segmentation finishes when PfCINCH localizes to the posterior end of the daughter cell. Residual PfCINCH forms relatively larger rings that enclose multiple nuclei inside one contiguous membrane as it progresses along budding cells in [−]ATc parasites. PfGAP45 staining is similar to that observed with PfMSP1 in these cells and demonstrates that the IMC has formed interior to the PPM, which surrounds the daughter cell aggregates (Fig. 3b).

To determine if other organelles were affected by PfCINCH knockdown, we used primary antibodies against the microneme protein PfEBA175, the rhoptry neck protein PfRON4, rhoptry bulb protein PfRhopH3, and trans-Golgi protein PfERD2. We also episomally expressed mCherry as a fusion protein to the mitochondrial targeting sequence from citrate synthase (PF3D7_1022500) and to the apicoplast targeting sequence from acyl carrier protein (PF3D7_0208500) to visualize mitochondria and apicoplast formation, respectively. Organelle formation was assessed in segmented schizonts, as defined by PfGAP45 co-staining surrounding daughter cells or by micronemal PfAMA1 co-staining. Aside from the morphological defects described above, no additional defects were identified with this panel of cell biologic markers, and unremarkable organelle staining was observed (Supplementary Fig. 5). These data demonstrate that PfCINCH-deficient parasites mature normally and form the required organelles and IMC. The critical failure for these mutant parasites is their aberrant contractile ring formation. Thus, they fail to direct the PPM around one nucleus and associated set of organelles, resulting in agglomerated daughter cells that progress through the egress trigger, but cannot reinitiate the asexual life cycle due to structural defects.

### PfCINCH-deficient parasites do not separate from residual body

**Fig. 4:**
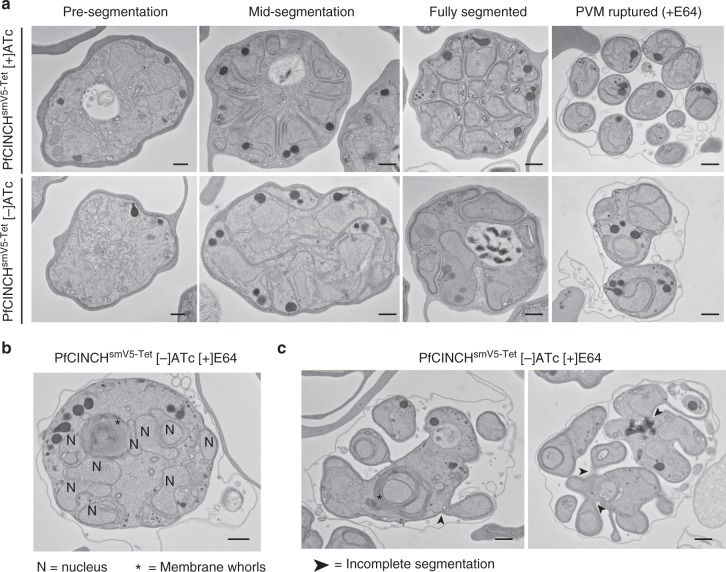
PfCINCH (*P. falciparum* *c*oord*i*nator of *n*ascent *c*ell detac*h*ment)-deficient parasites do not separate from the residual body. **a** Transmission electron micrographs of PfCINCH^smV5-Tet^ parasites [+] and [−]ATc over the course of segmentation. **b** Transmission electron micrograph of one PfCINCH-deficient schizont with nine nuclei and membrane whorls. **c** Transmission electron micrographs of PfCINCH-deficient schizonts containing incompletely segmented daughter cells and membrane whorls. **b** and **c** Capital n denotes nuclei, asterisks denote membrane whorls, and arrows point to incomplete segmentation of daughter cells. All scale bars 500 nm. SmV5, spaghetti monster V5; ATc, anhydrotetracycline

To examine the ultra-structure of PfCINCH-deficient parasites, we performed transmission electron microscopy (TEM). Purified 44 hpi parasites, 48 hpi parasites arrested with C1, and 52 hpi parasites arrested with the cysteine protease inhibitor E64 were prepared for TEM. E64 allows rupture of the parasite vacuolar membrane (PVM), but prevents RBC rupture, allowing visualization of merozoites that would have been released from the mother parasite^64^. As expected, PfCINCH^smV5-Tet^ parasites maintained on ATc segmented their daughter cells in an organized fashion, resulting in fully separated daughter cells (Fig. 4a). In PfCINCH-deficient parasites, however, we observed severe morphological defects. In mid-segmentation, daughter cell membranes formed around multiple nuclei and sets of organelles. Mature C1-treated PfCINCH-deficient daughter cells contained multiple sets of rhoptries and multiple nuclei, and the residual body was frequently engulfed by PPM in the center of a daughter cell agglomerate. These defects were highlighted in E64-treated parasites. Here we observed multiple sets of daughter cell material enclosed in one outer PPM (Fig. 4a). For example, a PfCINCH-deficient parasite with nine nuclei in one daughter cell is shown in Fig. 4b. Interestingly, we frequently observed large membrane whorls inside mutant cells (Fig. 4b, c). We were unable to identify their composition, but hypothesize that this is a consequence of abnormal development. We also observed daughter cells that remained connected to one another, suggesting that in addition to the segmentation defect, PfCINCH-deficient parasites fail to pinch off from the residual body at their basal ends (Fig. 4c). This hypothesis is supported by our time-lapse microscopy—after egressing, the majority of daughter agglomerates remained attached to one another as well as the residual body.

**Fig. 5:**
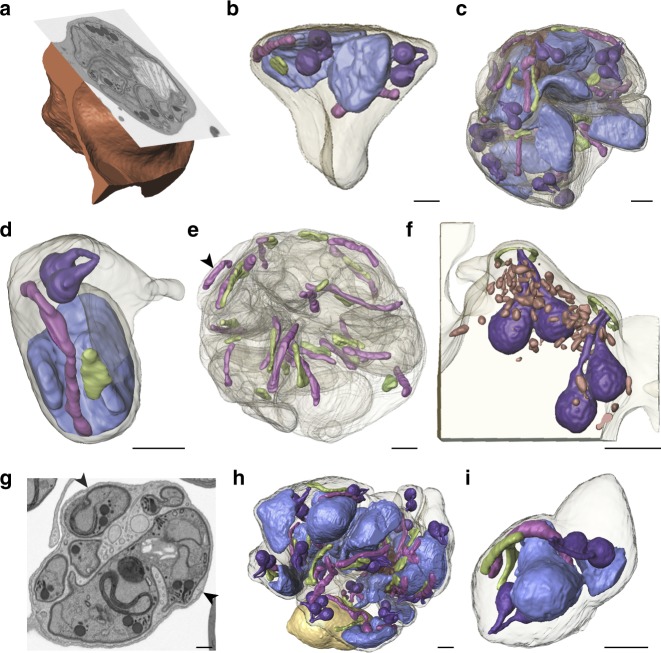
FIB-SEM of PfCINCH-deficient parasites **a** Ortho-slice of rendered red blood cell with SEM image of parasite. **b** Rendered fully segmented merozoite with two sets of nuclei (blue), apicoplasts (green), mitochondria (pink), and rhoptries (purple). **c** Rendered merozoite with 36 rhoptries, 18 apicoplasts, 19 mitochondria, and 11 nuclei. **d** Rendered merozoite with one set of organelles. **e** Rendered large merozoite membrane from **c** with only mitochondria and apicoplasts shown. Arrow identifies free mitochondrion with no apicoplast partner. **f** Apical end of a cross-section of one merozoite showing the apical ring (green), rhoptries (purple), and apical electron-dense organelles (red). **g** Representative SEM slice of mid- parasitophorous vacuolar membrane (PVM) rupture schizont. Arrows indicate PVM break. **h** Rendered schizont containing one large merozoite with transparent membrane and one small merozoite with shaded membrane. **i** Rendered small merozoite with transparent membrane. Scale bars in renderings interpolated from structure sizes in SEM micrographs. All scale bars 500 nm

As traditional TEM only allows for visualization of one 80–90 nm slice of the parasite, we used focused ion beam-scanning electron microscopy (FIB-SEM), a technique that allows for visualization and three-dimensional (3D) reconstruction of the entire parasite volume at the resolution of electron microscopy. We utilized FIB-SEM to examine E64-treated [+] and [−]ATc PfCINCH^smV5-Tet^ parasites pre-, during, and post-egress trigger. Unequivocal determination of parasite development was limited by the 1–2 h window of culture synchronization. Therefore, we focused on the status of the PVM to provide an objective measurement of parasite development within the selected 48–52 hpi window. Parasites with a fully intact PVM through all z-slices of the parasite were determined to be pre-egress trigger. Parasites where the PVM was mid-rupture, defined by direct visualization of PVM breaks in parasites with largely intact PVM, were categorized as mid-egress. Finally, parasites with full PVM disruption and loss of electron-dense material between individual merozoites were designated as post-egress trigger^64^. By evaluating the serial z-sections of PfCINCH-deficient parasites, we confirmed the defects observed by TEM, immunofluorescence microscopy, and time-lapse microscopy, but additionally observed that daughter merozoites indeed remain connected to one another at the basal end. We performed complete 3D reconstructions for two PfCINCH-deficient parasites, one prior to and one during egress. The pre-egress PfCINCH-deficient schizont contained merozoites of varying size—one merozoite contained components for two daughter cells, one contained two nuclei, apicoplasts and mitochondria, and eight rhoptries, and two daughter cells contained one set of rhoptries, one nucleus, one apicoplast, and one mitochondrion. The majority of parasite biomass, however, was within one large merozoite containing 11 nuclei, 18 apicoplasts, 19 mitochondria, 36 rhoptries, and the digestive vacuole (Fig. 5a–d and Supplementary Movie 3). This large merozoite contains more organelle sets than nuclei, likely because the final round of nuclear division is not yet complete in the pre-egress trigger schizont. Several of the merozoites remained attached to each other by a small membrane bridge, which we hypothesize is due to the pre-egress stage of the schizont as these structures can be observed in a [+]ATc schizont of a later stage (Supplementary Fig. 6a and d). Interestingly, we observed that despite the gross morphological defects in merozoite formation, apicoplasts and mitochondria divided and segregated relatively normally (Fig. 5e), in contrast to the TgMORN1 knockout where apicoplast division is impaired in *T. gondii*^43,44^. We also observed that the apical structure within these mutant parasite bulbs remained intact—throughout the volume of the parasite, rhoptries connect to the outer face of the cell and an apical ring structure is visible near each of the apices (Fig. 5f). Thus, while PfCINCH-deficient parasites are unable to properly define their boundaries or pinch off their membranes to separate from one another, they retain the polar arrangement of wild-type parasites. The 3D reconstruction of the mid-egress PfCINCH-deficient schizont revealed two merozoites—a small daughter cell contained one nucleus, four rhoptries, one mitochondria, and two apicoplasts, while a large daughter cell contained 22 nuclei, 53 rhoptries, 25 apicoplasts, 27 mitochondria, and the food vacuole. (Fig. 5g–i) The ratios of nuclei to sets of organelles vary in this parasite, but for the most part organelles and nuclei are divided, suggesting that the PfCINCH-dependent function of the basal complex is independent from nuclear and organellar division. Finally, reconstruction of the mid-egress schizont grown in the presence of ATc revealed, as expected, 24 individual merozoites each with a single nucleus, apicoplast, mitochondrion, and apical end with a single set of rhoptries (Supplementary Fig. 6). The narrow membranous connections observed between merozoites in the pre-egress and mid-egress parasites, for both [+]ATc and [−]ATc parasites, become fully severed in the post-egress parasites (Supplementary Movies 4 and 5). Therefore, by performing FIB-SEM, we elucidated that PfCINCH is required for establishing an appropriately sized contractile ring and the consequent production of daughter merozoites with appropriately partitioned organelles.

### Immunoprecipitation of PfCINCH-containing complexes

**Fig. 6:**
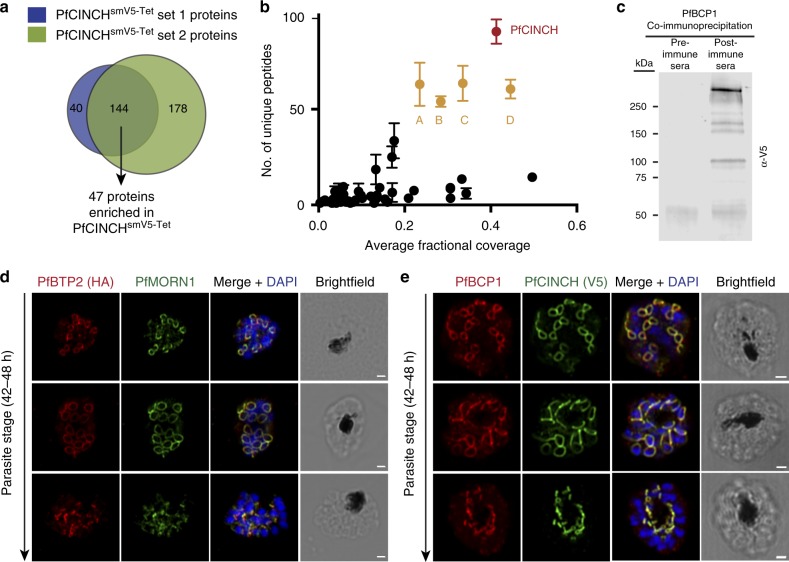
Immunoprecipitation of PfCINCH (*P. falciparum* *c*oord*i*nator of *n*ascent *c*ell detac*h*ment)-containing complexes. **a** Overlap of proteins present in two biological replicates of the PfCINCH immunoprecipitation that were not present in the control. **b** Graph of the average fractional coverage and number of unique peptides identified for proteins present in both PfCINCH immunoprecipitation experiments. Mean ± SD for two replicates shown. A: PF3D7_0704100/PfBTP2 (*P. falciparum* *b*asal complex *t*ransmembrane *p*rotein 2), B: PF3D7_1436200/PfBCP1 (*P. falciparum* *b*asal *c*omplex *p*rotein 1), C: PF3D7_1018200, and D: PF3D7_1351700. **c**, Immunoblot of eluate from PfBCP1 co-immunoprecipitation, probed with α-V5. Representative blot from two biological replicates. **d** Airyscan super-resolution micrographs of PfBTP2 and PfMORN1 throughout segmentation. **e** Airyscan super-resolution micrographs of PfBCP1 and PfCINCH throughout segmentation. All scale bars 1 µm. Source data are provided as a Source Data file

Aside from PfMORN1, PfBTP1, PfHAD2, and now, PfCINCH, other components of the *Plasmodium* basal complex have not been identified. To discover these proteins, we immunoprecipitated PfCINCH^smV5^-containing complexes. As a control, we immunoprecipitated PF3D7_1419700^smV5^, a cytosolic translation initiation factor, in a parallel transgenic strain (Fig. 6a). In two biological replicates, PfCINCH was the most highly represented protein, with 95 and 86 unique peptides, respectively, identified (Fig. 6a, b). We defined top hits as proteins with > 50 unique peptides in each pulldown, >20% protein coverage, and exclusive presence in the PfCINCH pulldown. Using these criteria, four proteins were identified as top hits (Fig. 6b and Supplementary Data 1). These include PF3D7_1351700 (PfIMC1f), PF3D7_1018200 (PfPPP8), PF3D7_0704100 (conserved *Plasmodium* membrane protein of unknown function), and PF3D7_1436200 (conserved *Plasmodium* protein of unknown function). Just below these strict thresholds, we also identified Pf3D7_1229800/PfMyoJ (40/27 unique peptides, 17.5% coverage, 0 peptides in control). PfIMC1f and PfMyoJ both have *Toxoplasma* orthologs, TgIMC13 and TgIMC15 for PfIMC1f^34^, and TgMyoJ for PfMyoJ^52^. Both TgIMC13 and TgMyoJ are members of the *Toxoplasma* basal complex, while TgIMC15 localizes to both the apical cap and basal end of the budding daughter cell.

To validate our PfCINCH^smV5^ immunoprecipitation results, we performed a reciprocal pulldown with primary antisera against PF3D7_1436200 in PfCINCH^smV5-Tet^ parasites and probed for PfCINCH^smV5^ in the eluate by immunoblot. PfCINCH^smV5^ was detectable in the immunoprecipitation with the antisera, but not with pre-immune sera, validating our mass spectrometry results (Fig. 6c). PF3D7_1436200 is a 270 kDa *Plasmodium-*specific protein with maximal expression at 38 hpi with no predicted domains or transmembrane domains^2^. Therefore, we propose to name this protein basal complex protein 1 (PfBCP1). While the anti-PfBCP1 antiserum was functional for immunoprecipitation and immunofluorescence (Fig. 6c and e, respectively), it does not work well for immunoblotting, and we are therefore unable to evaluate the amount of PfBCP1 that was pulled down. PF3D7_0704100 is a *Plasmodium*-specific (among Apicomplexa), 425 kDa protein with maximal expression at 38 hpi^2^. It has seven predicted transmembrane domains at its N-terminal end, but no other identifiable domains. Following the convention of PfBTP1 naming^45^, we name PF3D7_0704100 basal complex transmembrane protein 2 (PfBTP2). We fused a triple HA epitope tag to the carboxy terminus of PfBTP2 in wild-type 3D7 parasites (Supplementary Fig. 7). We confirmed the localization of PF3D7_1436200 and PF3D7_0704100, the two most abundant proteins of unknown function in our dataset. For PfBTP2, we performed immunofluorescence analysis on 44–48 hpi schizonts by co-staining parasites with α-HA antibodies (recognizing PfBTP2) and α-PfMORN1 primary antisera. In early schizonts, PfBTP2 and PfMORN1 form small rings with overlapping fluorescent signals. As segmentation progresses, both PfBTP2 and PfMORN1 move along the outer edge of nascent cells, forming larger rings at the widest point that condense as they reach the basal end of the parasite. Finally, when segmentation is complete, we observe punctate PfBTP2 and PfMORN1 staining at the posterior end of the parasite (Fig. 6d). For PfBCP1, we used the primary antisera against PfBCP1 to localize this protein in PfCINCH^smV5-Tet^ schizonts. Similarly, we performed immunofluorescence assays on 46–48 h schizonts and observed that PfBCP1 overlaps with PfCINCH throughout segmentation, confirming that it is a novel member of the *Plasmodium* basal complex (Fig. 6e). These results confirm that our PfCINCH^smV5^ pulldown resulted in the identification of bona fide novel basal complex proteins with previously unknown localization, including one transmembrane protein, PfBTP2, and one protein without known domains, PfBCP1.

## Discussion

Parasites deficient in PfCINCH have multiple morphological defects. First, they form agglomerates that contain the components for several daughter cells within one PPM. Following knockdown, the PfCINCH-deficient parasites contain residual PfCINCH, but this level is below a critical level that is required to form proper invasive merozoites. We could not discern a consistent pattern for how many nuclei were engulfed in a single PPM and observed variable sizes of daughter cells. We hypothesize that this range is likely due to uneven distribution of the remaining PfCINCH protein, as in our IFAs (immunofluoresence analyses) we observe residual PfCINCH^smV5^ staining.

PfCINCH-deficient parasites, in addition to failing to segment their daughter cells, also fail to pinch these agglomerates off from one another. We observed this defect by time-lapse microscopy and were able to visualize connected parasite bulbs by TEM. Using FIB-SEM, however, we were able to more robustly observe this phenotype in multiple mutant parasites. This defect is similar to the one observed by Lorestani et al.^43^ and Heaslip et al.^44^ in a TgMORN1 knockout. ΔTgMORN1 parasites were able to fully separate their daughter cells, but these could not pinch off from the residual body at their basal ends.

Interestingly, despite these severe morphological defects, PfCINCH-deficient cells still responded, apparently normally, to the poorly characterized egress trigger. This result suggests that, even following aberrant cytokinesis, parasites can egress from the RBC. It is formally possible that parasites completely deficient in PfCINCH would arrest at an earlier stage of parasite development and that residual PfCINCH allows for the development of parasites, albeit morphologically aberrant parasites, to the egress trigger. This possibility will be explored in future work in a parasite line with more robust PfCINCH depletion or inducible knockout. Despite these clear phenotypes, it is unresolved whether PfCINCH is directly responsible for maintaining the integrity of the contractile ring and pinching off daughter cells, or if knockdown of PfCINCH results in the disintegration of the basal complex, thus deserting the proteins responsible for these functions.

Finally, we leveraged our PfCINCH^smV5-Tet^ parasite line to identify additional basal complex members. First, we confirmed that PfBCP1 and PfBTP2 are novel basal complex members. OrthoMCL identifies PfBTP2 as an ortholog of the yeast protein Num1, which is responsible for anchoring dynein to the forming cortical bud, thereby allowing DNA migration into the daughter cell^65,66^. This implies a putative role for PfBTP2 in segmentation. We also identified a serine/threonine phosphatase; PfPPP8 that contains a calcium-binding EF hand. PfPPP8 may act as a modulator of signaling pathways that converse with the basal complex. PfIMC1f, an ortholog of the *T. gondii* basal complex proteins TgIMC13 and TgIMC15^34^, and PfBCP1, a conserved protein of unknown function, were also identified as top hits. We set a stringent cutoff for top hits to avoid false positives; however, many proteins below this threshold may also be involved in basal complex or IMC function. For example, PfMyoJ, the ortholog of the recently identified essential *T. gondii* basal complex myosin, TgMyoJ^52^, fell just below the cutoff. Furthermore, both PfBTP1 and PfMORN1 were among our top 20 hits in both biological replicates. Therefore, proteins identified as frequently as PfMORN1 and PfBTP1 should be investigated for their potential role in this complex.

In conclusion, we have defined the role of a conserved *Plasmodium* protein, PF3D7_0407800 (PfCINCH), with no previously known function. We used several cell biological techniques to localize PfCINCH to the basal complex, examine morphological defects in PfCINCH-deficient parasites, and confirm the proper development of organelles in mutant parasites. Finally, we immunoprecipitated PfCINCH complexes to identify novel basal complex proteins. This work provides a launch pad from which we can further investigate the myriad proposed basal complex functions, tie functions to specific proteins, and determine the mechanisms by which basal complex proteins accomplish these functions.

## Methods

### *Plasmodium falciparum* culturing and transfection

The 3D7 strain of *P. falciparum*, obtained from the Walter and Eliza Hall Institute (Melbourne, Australia) was used for all assays. Parasites were cultured in RPMI-1640 (Sigma) supplemented with 25 mM HEPES (4-(2-hydroxyethyl)-1-piperazineethanesulfonic acid) (EMD Biosciences), 0.21% sodium bicarbonate (Sigma), 50 mg/l hypoxanthine (Sigma), and 0.5% Albumax (Invitrogen). Packed RBCs were obtained from Valley Biomedical.

For CRISPR transfection, approximately 100 μg of HDR plasmid was linearized by digestion, purified, and co-transfected with 100 μg Cas9 targeting plasmid into 3D7 parasites. Parasites transfected with the Tet aptamer system were maintained on 500 nM ATc. One day post transfection, drug pressure was applied with 2.5 nM WR99210 (Jacobus Pharmaceuticals) and the PfDHODH inhibitor *N*-(3-chloro-4-methylphenyl)-5-methyl-2-(trifluoromethyl)[1,2,4]triazolo[1,5-a]pyrimidin-7-amine (MMV665874 or AD1) at 150 nM. Five days after transfection, AD1 selection was removed. For episomal plasmid transfection, approximately 100 μg of plasmid was transfected, and drug pressure was applied from the day after transfection on. Transgenic parasites were cloned by limiting dilution and integration of the targeting construct for PfCDPK5 was confirmed by PCR with oligos oJDD2679/oJDD2441 (control), oJDD1586/oJDD1409 (integration), and oJDD1586/oJDD1350 (locus size). For PfCINCH, oligos oJDD3924/oJDD3925 (control), oJDD4070/oJDD2933 (integration), and oJDD4070/oJDD3923 (locus size) were used. Tet aptamer size was confirmed by amplifying the aptamer with oligos oJDD3560/oJDD44 and digesting the PCR fragment with *Psp*OMI and *Kpn*I. All sequences for oligonucleotides are provided in Supplementary Data 2.

### Reagents and antibodies

Primers were obtained from Integrated DNA Technologies or Life Technologies; restriction enzymes were obtained from New England Biolabs. Commercially available antibodies were obtained from Sigma (rat anti-HA, clone 3F10), Chromotek (rat anti-RFP, clone 5F8), and Bio-Rad (mouse anti-V5, clone SV5-Pk2). Other antibodies were kindly provided by Robin Anders at The Walter & Eliza Hall Institute of Medical Research (mouse anti-PfAMA1 clone 1FG), Alan Cowman, Jenny Thompson, and Kaye Wycherley at The Walter & Eliza Hall Institute of Medical Research (mouse anti-PfRON4)^67^, Julian Rayner at Wellcome Trust Sanger Institute (rabbit anti-PfGAP45)^68^, Anthony Holder at MRC National Institute for Medical Research (mouse anti-PfMSP1, clone 1E1)^62^, Odile Puijalon at Institut Pasteur Paris (mouse anti-PfRophH3)^69^, and Michael Makler at Flow Inc. (mouse anti-PfLDH). Rabbit anti-ERD2 (MRA-1)^70^ and mouse anti-PfEBA175^71^ (R218 antibody produced from hybridoma, MRA-712) were obtained through the Malaria Research and Reference Reagent Resource Center as part of the BEI resources, National Institute of Allergy and Infectious Diseases (NIAID), National Institutes of Health (NIH), contributed by John Adams and EntreMed/NIAID, respectively.

### Plasmid construction

To generate a PfCDPK5 targeting plasmid, the *P. falciparum* U6 promoter was amplified from 3D7 genomic DNA (gDNA) with oJDD2580/oJDD2814 and oJDD2583/oJDD2815 and cloned into an *Eco*RI site in pSAB01^5^ with *Eco*RI and *Mfe*I to generate pRR52. The 3′ end of PfCDPK5 was codon altered (caPfCDPK5) by gene block synthesis (gBlock from IDT DNA Technologies, full sequence in Supplementary Data 2) and amplified with oJDD2681/oJDD2682, and the 500 bp preceding coPfCDPK5 was amplified with oJDD2679/oJDD2680 from gDNA. PCR splicing by overhang extension (SOE) was utilized to fuse the 5′ HR with coCDPK5 and the piece was cloned into pRR52 with *Not*I and *Xho*I to generate pRR53. The PfCDPK5 3′ HR was amplified from gDNA with oJDD2683/oJDD2684 and cloned into pRR53 with *Eco*RI and *Pst*I to generate pRR57. To create a repair plasmid that would fuse nLuc and 3HA onto PfCDPK5 in a marker-free fashion, we amplified nanoluciferase with oJDD3321/oJDD3322 from pNluc1.1 (Promega) and the PfCDPK5 3′ HR with oJDD3321/oJDD3320. We used PCR SOE to fuse the fragments and cloned them into pRR57 with *Xho*I/*Avr*II to generate pRR61.

We used pUF1-Cas9^72^ to engineer a Cas9 targeting plasmid. We introduced the *P. falciparum* U6 cassette containing a U6 promoter, *Bbs*I sites for guide cloning, the scaffold guide RNA, and U6 terminator. Oligos oJDD3036/oJDD3037 were used to amplify the U6 promoter and oJDD3038/oJDD2789 were used to amplify the guide RNA and U6 terminator from pRR57 with newly introduced *Bbs*I sites to subsequently clone gene-specific guides. These fragments were fused by PCR SOE and cloned into pUF1-Cas9 with *Eco*RI and *Avr*II to generate pBAM202. We next amplified the rep20 sequence^73^ with oligos oJDD3039/oJDD3040 and cloned into a *Not*I site in pBAM203. PfCDPK5- and PfCINCH-targeting guides were cloned into the U6 cassette by PCR SOE. The U6 promoter with guide was amplified by oJDD3058/oJDD2702 and oJDD3058/oJD3927 and gRNA and U6 terminator with guide amplified with oJDD3059/oJDD2701 and oJDD3059/oJDD3928 for PfCDPK5 and PfCINCH, respectively. The cassettes were cloned into pBAM203 with *Eco*RI and *Avr*II to generate pRR63 for PfCDPK5 and pRR99 for PfCINCH.

To create a plasmid for fusing the smV5 tag and the tet aptamer system to genes of interest, we first synthesized a gene block with 3HA-MCS (multiple cloning site), (gBlock from Integrated DNA Technologies) with three HA epitope tags and an MCS, and subcloned it into pSAB01 with *Not*I and *Kpn*I to generate pVAS70^5^. Next, we subcloned the 10X-Tet Aptamer from pMG62^56^ into the MCS with *Apa*I and *Xma*I (pVAS71). To facilitate future cloning, we added a second MCS containing *Avr*II, *Cla*I, *Bsi*WI, and *Pst*I using oligos oJDD3541/oJDD3542 to an *Eco*RI site in the plasmid, generating pRR65. To add TetR-DOZI to the hDHFR cassette, we amplified TetR-DOZI from pMG62 with oligos oJDD3481/oJDD3253, the Pfhsp86 promoter with oligos oJDD3480/oJDD3482, and the coding sequence for hDHFR and the PfhrpII terminator with oligos oJDD3254/oJDD3483. We then used PCR SOE to generate an hsp86-TetR-DOZI-2A-hDHFR-hrpII cassette where TetR-DOZI and hDHFR are joined by the 2A ribosomal skip peptide. We cloned this cassette into pRR65 with *Afl*II and *Avr*II (pRR67). To add smV5^54^ for 3′ tagging, we amplified the coding sequence from pCAG_smFP-V5 (a gift from Loren Looger, Addgene plasmid #59758) with oligos oJDD3484/oJDD3485 and cloned into pRR67 with *Nco*I/*Psp*OMI to generate pRR69.

To prevent homology directed repair from occurring between the Cas9-directed cut site and the 3′ end of PfCINCH, we synthesized a gene block (IDT DNA Technologies) caPfCINCH where the last 966 bp of PfCINCH are codon altered. We amplified this gene block with oligos oJDD3941/oJDD3942, and the 500 bp preceding the codon altered region from 3D7 gDNA with oligos oJDD3924/oJDD3926. We amplified 500 bp from the 3′ end of PfCINCH for the 3′ homology region with oligos oJDD3922/oJDD3923 from 3D7 gDNA. We again used PCR SOE to create a PfCINCH 3′HR-*Eco*RV-*Stu*I-*Sac*II-PfCINCH 5′HR-caPfCINCH fusion. We cloned this fusion into pRR69 with *Not*I and *Nco*I to generate pRR92.

PF3D7_0704100 (PfBTP2) was targeted with a vector for 3′ homologous recombination. We amplified the PF3D7_0704100 homology region from 3D7 gDNA with oJDD1551/oJDD1552 and cloned into a plasmid for 3′ end tagging with 3HA-DD-glmS with *Not*1/*Xho*I to generate pVAS29.

To generate the mitochondria-targeted mCherry, the leader sequence mitochondrial citrate synthase (PF3D7_1022500)^9^ was amplified from 3D7 gDNA with oJDD4322/oJDD4323 and cloned into *Xho*I/*Kpn*I of p1xNLS-FRB-mCherry with nmd3-promoter yDHODH^74^ to create pJPM21. The apicoplast-targeted mCherry plasmid, pJPM29, was cloned by amplifying the leader sequence from the acyl carrier protein (PF3D7_0208500) with oJDD4320/oJDD4321 and cloning it in-frame with mCherry in a yDHODH-containing plasmid where the transgene is expressed from the Pfhsp86 promoter.

### PfCINCH depletion

In all PfCINCH depletion assays, PfCINCH^smV5-Tet^ and control strain schizonts were purified by density centrifugation with 60% Percoll, washed twice in ATc-free RPMI, and allowed to reinvade fresh erythrocytes for 2–3 h in the absence of ATc. New rings were purified by 5% (w/v) sorbitol synchronization and washed two additional times in ATc-free RPMI. Isolated rings were split 1:2 with half of the synchronized parasites added to RPMI + 500 nM ATc, and half added to RPMI + 0 nM ATc.

### Growth assays

Synchronized, PfCINCH^smV5-Tet^ and parental parasite lines were diluted to 0.2% parasitemia at 1% hematocrit. One hundred microliters of culture was collected on days 1, 3, 5, and 7 and immediately fixed in 1% paraformaldehyde (PFA) in Alsever’s solution. Fixed cells were washed in Alsever’s, resuspended in 1:1000 dilution of SYBR green I (Invitrogen) in 0.5% bovine serum albumin-phosphate buffered saline (BSA-PBS), and incubated for 20 min at room temperature. Stained parasites were washed in 0.5% BSA-PBS and resuspended in filtered PBS. The proportion of infected cells was measured by flow cytometry. For MFI determination, parasites were fixed in PBS with 2% PFA and 0.2% glutaraldehyde, permeabilized in 0.3% Triton-X 100 for 10 min at room temperature, incubated with 0.5 mg/mL RNAse-PBS for 1 h at 37 °C, and stained as above.

### Quantitative western blotting

Parasite pellets were purified by lysing RBCs in 0.05% saponin in PBS with protease inhibitors (SigmaFast Protease Inhibitor Cocktail, Sigma) and boiled in Laemmli buffer. The equivalent of 1 mL of parasites at 1% schizontemia was run on a 4–20% Tris-glycine-sodium dodecyl sulfate gel and transferred to a polyvinylidene fluoride membrane. Membranes were blocked in Licor Odyssey blocking buffer, incubated with primary antibody diluted (1:1000 for anti-V5 or 1:2000 for anti-PfLDH) in blocking buffer, and then incubated with secondary antibodies diluted in blocking buffer. Membranes were scanned on a Licor Odyssey CLx imager system and quantified using volumetric measurement of fluorescence intensity. Uncropped western blot images are provided in the Source Data file.

### Generation of primary antisera

Full-length PfMORN1 was synthesized as a codon-optimized gene block (coPfMORN1), PCR amplified with oJDD3034/3035, and cloned into pET28b with *Nde*I and *Xho*I. The N-terminal 6-His tagged recombinant protein was expressed in *E. coli*, purified on Ni-NTA resin as per the manufacturer’s recommendation, and used for generation of polyclonal antisera from rabbits (Cocalico Biologicals Inc.). Polyclonal rabbit antisera against amino acids 471–484 of PF3D7_1436200/PfBCP1 (KNDQNGENDDIRKN-C) conjugated to keyhole limpet hemocyanin was generated and affinity purified (Genscript).

### Immunofluorescence assays

Dried blood smears were fixed with 4% PFA for 10 min. Following fixation slides were washed 3× in 1× PBS, parasites permeabilized with 0.1% Triton X-100 in 1× PBS for 10 min, and washed 3× in 1× PBS. Slides were blocked in 3% (w/v) BSA in 1× PBS for 1 h at room temperature or overnight at 4 °C. Primary antibodies were diluted in blocking solution and slides were incubated with primary for 1 h at room temperature or overnight at 4 °C. Slides were washed 3× in 1× PBS and secondary antibodies were diluted in 0.5% BSA-PBS and applied for 45 min at room temperature. Slides were washed 3× and coverslips were mounted with VectaShield Hard Mount with DAPI (4′,6-diamidino-2-phenylindole). Cells were visualized on a Zeiss LSM700 for confocal microscopy or a Zeiss LSM880 with Airyscan for super-resolution microscopy. Dilutions for primary antibodies were as follows (mouse anti-PfRON4 1:200, mouse anti-V5 1:200, rabbit anti-PfGAP45 1:5000, rabbit anti-PfMORN1 1:1000, mouse anti-PfMSP1 1:500, mouse anti-PfAMA1 1:200, rat anti-HA 3F10 1:50, rabbit anti-PfBCP1 1:2500, mouse anti-PfRhopH3 1:1000, mouse anti-PfEBA175 1:500, rat anti-RFP 1:1000, rabbit anti-PfERD2 1:200).

### Time-lapse microscopy

Synchronized PfCINCH^smV5-Tet^ parasites [+]/[−]ATc were obtained as described above. For C1 washout experiments, at 44 hpi, schizonts were magnetic-activated cell sorting (MACS) purified and then incubated with 2.5 μM C1. At 48 hpi, parasites from each condition (one at a time) were washed with C1-containing or C1-free media, 25 μL applied to the inside of a frame-seal incubation chamber (Bio-Rad), and sealed with a coverslip. Ten minutes post washout, parasites were recorded at 1 fps for 15 min. For egress visualization without C1, 48-h parasites were MACS purified and allowed to settle on conconavalin A-coated glass bottom dishes and egress events were recorded at 1 fps. Several different fields were monitored over 3 h. Parasites were visualized on a Nikon TiEclipse or Zeiss AxioObserver.

### Transmission and FIB-SEM

Synchronized PfCINCH^smV5-Tet^ parasites [+]/[−]ATc were MACS column purified at 44 hpi. One-third of parasites for each condition were treated with 1 mM E64 and one-third were treated with 2.5 μM C1. Remaining parasites were resuspended in fixative (2.5% paraformaldehyde, 5% glutaraldehyde, 0.06% picric acid in 0.2 M cacodylate buffer) at 44 hpi. C1-treated parasites were fixed at 48 hpi and E64-treated parasites were fixed at 52 hpi. Fixed parasites were subjected to standard TEM preparation and visualized on a JEOL 1200EX. FIB-SEM data for [−]ATc PfCINCH^smV5-Tet^ samples were recorded on a Zeiss Crossbeam 550 operating at a voltage of 1.5 kV and a current of 0.8 nA using the Atlas control software and the energy-selective backscatter detector. The reference samples containing [+]ATc PfCINCH^smV5-Tet^ parasites were recorded at the Center for Nanoscale Systems using a FEI Helios Nanolab 660, operated in immersion mode at 2 kV/1.6 nA, and controlled via the Auto Slice & View 4 software package, using an in-column backscatter detector. The staining procedure for TEM samples produced sufficient contrast for reliable data segmentation, which allowed use of the same sample blocks for the FIB-SEM analysis by mounting onto a standard SEM stub and rendering conductive via 5–10-nm-thick sputter-coated Pt/Pd film, leaving the face that was produced during TEM sample preparation as the top-most surface. An appropriated region of interest was selected by imaging with a backscatter detector and then protected using a 1–2-μm-thick platinum film of size 26 × 26 μm^2^ deposited via ion-beam-assisted decomposition of a Pt-containing gas. After preparing the appropriate fiducial markers and trenching out the material to a visual depth of 20 μm in front of the region of interest with a high ion beam current, the final slicing was done at an ion voltage of 30 kV and 800 pA beam current. In either case, the recorded 3D volumes contained stacks of images with a pixel size of approximately 5 nm and a slice thickness of 20 nm. Images were aligned via cross-correlation, denoised with the non-local means filter, and binned using an in-house Matlab-based software package leading to volumes with a voxel size of 10 × 10 × 20 nm^2^. Data segmentation and visualization was performed using Avizo 9.2.

### Immunoprecipitation assays

Experimental and control parasite lines were tightly synchronized by density centrifugation with 60% Percoll, allowed to reinvade fresh erythrocytes for 2–3 h, and new rings were isolated with 5% (w/v) sorbitol. Synchronized parasites were expanded to 200 mL at 2% schizontemia per condition and collected at approximately 48 hpi. At collection, RBCs were lysed with 0.05% saponin in PBS with protease inhibitors (SigmaFast Protease Inhibitor Cocktail, Sigma). Parasite pellets were lysed in RIPA (50 mM Tris-HCl, pH 7.5, 150 mM NaCl, 1% NP-40, 0.5% sodium deoxycholate, 0.1% sodium dodecyl sulfate) plus protease inhibitor for 30 min on ice, sonicated twice at 20% amplitude for 30 s, and insoluble material was removed by centrifugation. Parasite lysate was applied to magnetic α-V5 beads (MBL International) and incubated for 3 h at 4 °C. Beads were washed three times with fresh RIPA plus protease inhibitors, resuspended in 50 μL of 5 mM ammonium bicarbonate, and submitted for detergent removal, on-bead digestion, and mass spectrometry analysis. Mass spectrometry results were analyzed by comparing the number of unique and total peptides present in experimental and control samples. For co-immunoprecipitation with PF3D7_1436200 (PfBCP1) antisera, 70 mL Percoll-sorbitol synchronized PfCINCH^smV5-Tet^ schizonts at 2% parasitemia were collected and lysed as above. Parasite lysates were pre-cleared with Protein A beads (Life Technologies Dynabeads Protein A) for 1 h at 4 °C. Pre-cleared lysate was separated from beads and divided into two fractions; to one fraction 2.5 μg of PfBCP1 antisera was added, and to the other 5 μL pre-immune sera from the same rabbit was added. Lysates and sera were incubated for 1 h at 4 °C, then Protein A beads were added for an additional hour with rotation. Beads were washed three times in RIPA and samples eluted by boiling in Laemmli buffer. Eluates were analyzed by immunoblotting as described above.

### Reporting summary

Further information on research design is available in the Nature Research Reporting Summary linked to this article.

## Supplementary information

Supplementary Information

Reporting Summary

Description of Additional Supplementary Files

Supplementary Data 1

Supplementary Data 2

Supplementary Movie 1

Supplementary Movie 2

Supplementary Movie 3

Supplementary Movie 4

Supplementary Movie 5

Source Data

## Data Availability

All relevant data are available from the authors upon request. The source data underlying Figs. 2a–d and 6c, and Supplementary Figs. 1a–d, 2, 3a–c, and 4b, c are provided as a Source Data file.
