# Supplementary material for: An essential contractile ring protein controls cell division in Plasmodium falciparum
Source: Nat Commun. 2019 May 16;10:2181. doi: 10.1038/s41467-019-10214-z (PMC6522492; doi:10.1038/s41467-019-10214-z)
Supplement: Supplementary file 1 — Supplementary Information [file 41467_2019_10214_MOESM1_ESM.pdf]

Supplementary Information

An essential contractile ring protein controls cell division in *Plasmodium falciparum*

Rudlaff, et al

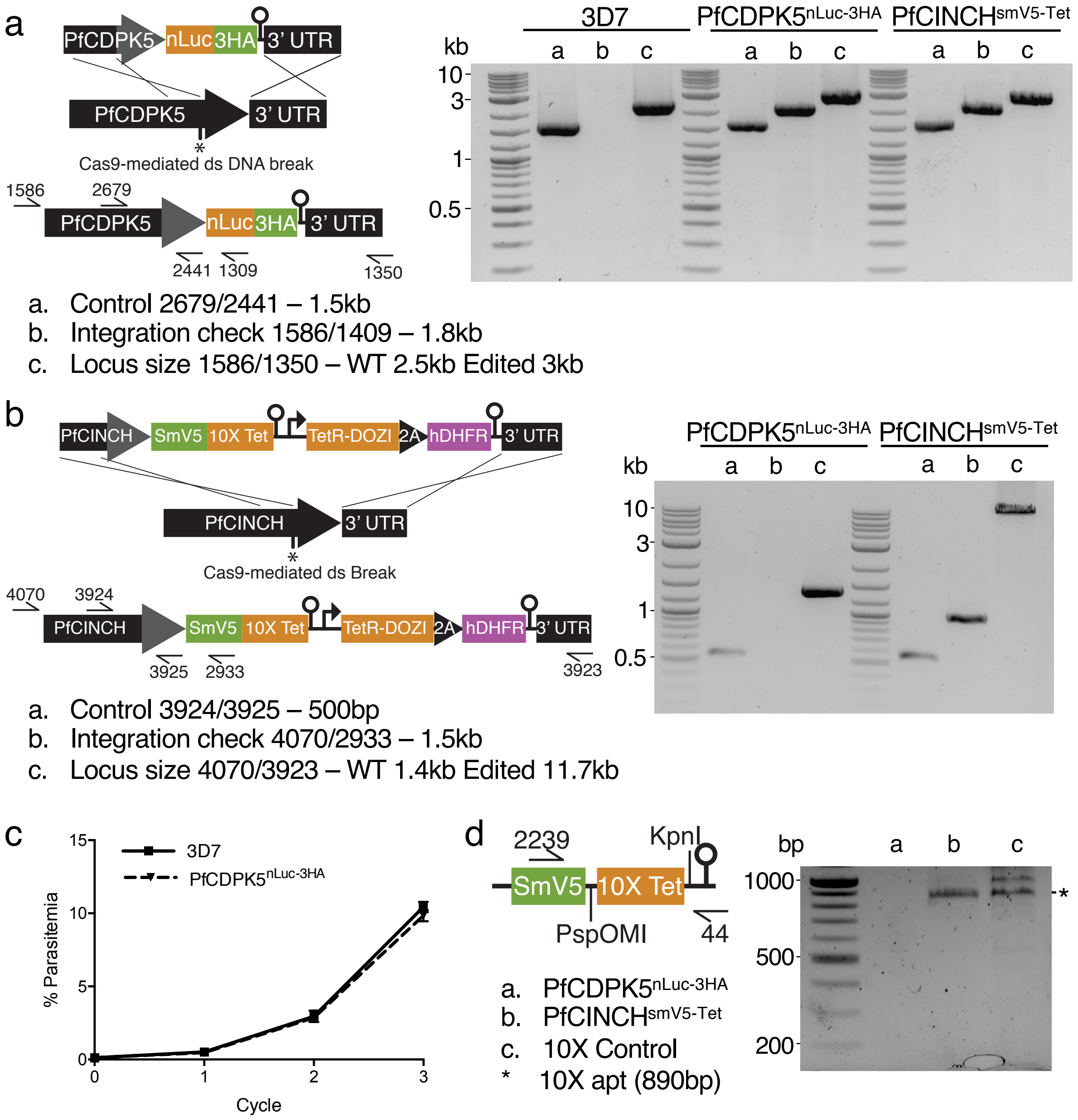

Supplementary Figure 1: Generation of the PfCINCH$^{smV5\text{-}Tet}$ parasite line. a, Generation of the transgenic "Parental" line where PfCDPK5 is fused to 3HA-nLuc. **b,** Generation of the PfCINCH$^{smV5\text{-}Tet}$ transgenic parasite line. **c,** The PfCDPK5$^{3HA\text{-}nLuc}$ parasite strain has similar growth kinetics to the wild-type 3D7 parasite strain. Error bars (mean +/- SD of triplicate wells) are too small to be visible. **d,** Ten copies of the Tet aptamer are present in the PfCINCH$^{smV5\text{-}Tet}$ parasite line. Source data are provided in the Source Data file.

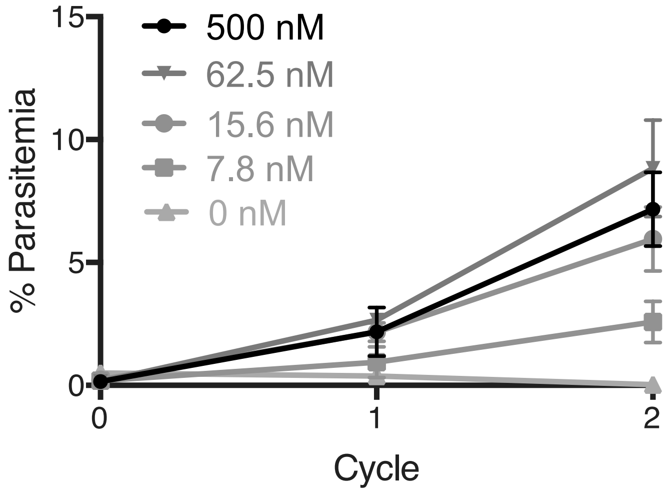

Supplementary Figure 2: Titration of ATc concentration results in differential PfCINCH$^{smV5\text{-}Tet}$ parasite growth. Percent parasitemia of PfCINCH$^{smV5\text{-}Tet}$ parasites over two replication cycles at different ATc concentrations shown. Parasitemia was determined by counting 100 cells per replicate (3n) per condition. Error bars mean +/- SD. Source data are provided in the Source Data file.

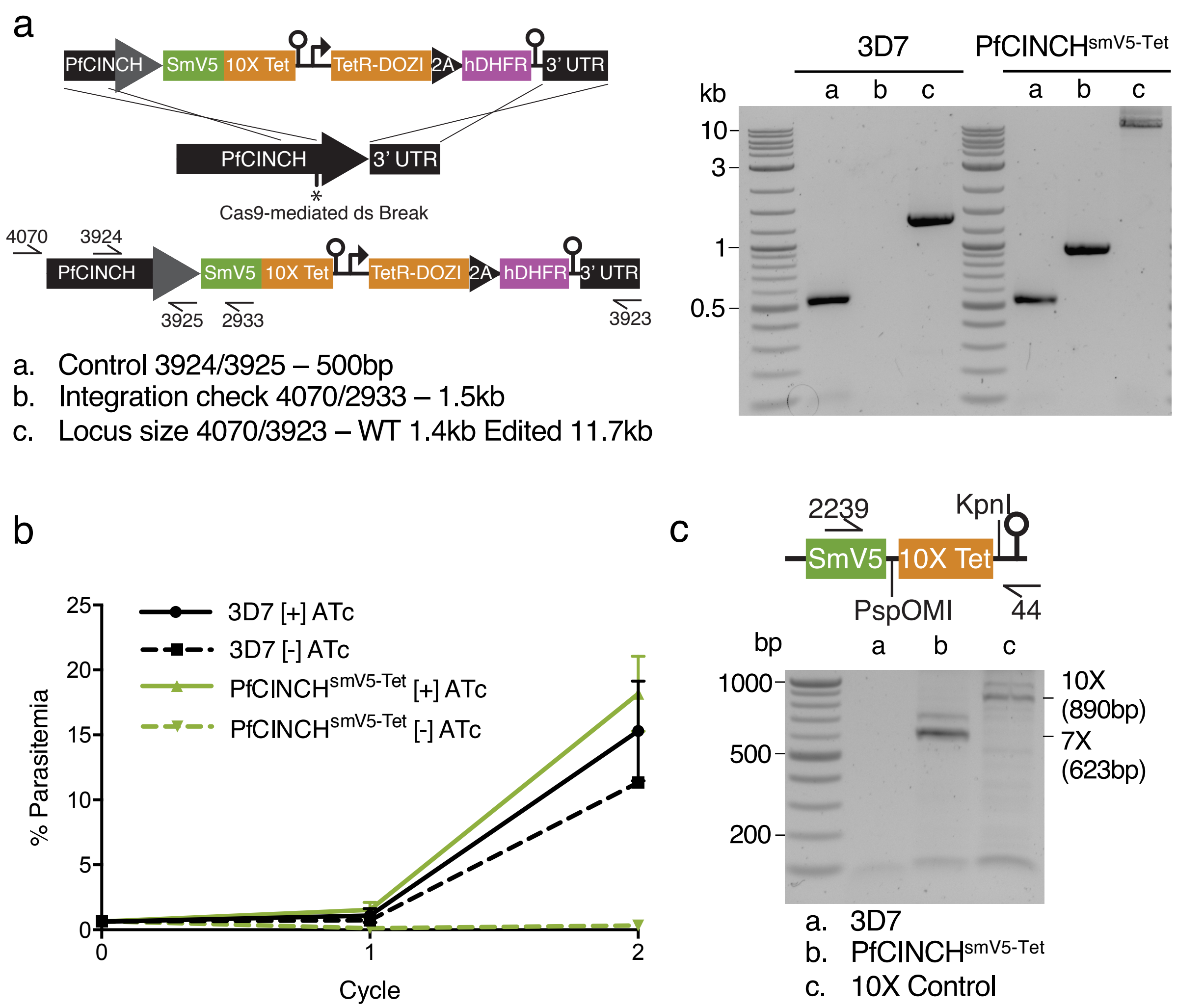

Supplementary Figure 3: Generation of a PfCINCH$^{smV5\text{-}Tet}$ transgenic parasite line in a 3D7 background. a, Schematic for targeting construct integration into the parasite genome and expected sizes for PCRs from 3D7 and transgenic lines. Agarose gel demonstrating integration of the PfCINCH targeting construct into the locus and concomitant increase in locus size. **b,** Growth curve of PfCINCH$^{smV5\text{-}Tet}$ transgenic parasites [+]/[-]ATc. Parasitemia for cycles 0 and 2 was measured by flow cytometry. Parasitemia for cycle 1 was determined by counting 200 cells per condition per replicate. Mean +/- SD of triplicate wells shown. **c,** The PfCINCH$^{smV5\text{-}Tet}$ parasite line contains a 7X Tet aptamer. Source data are provided in the Source Data file.

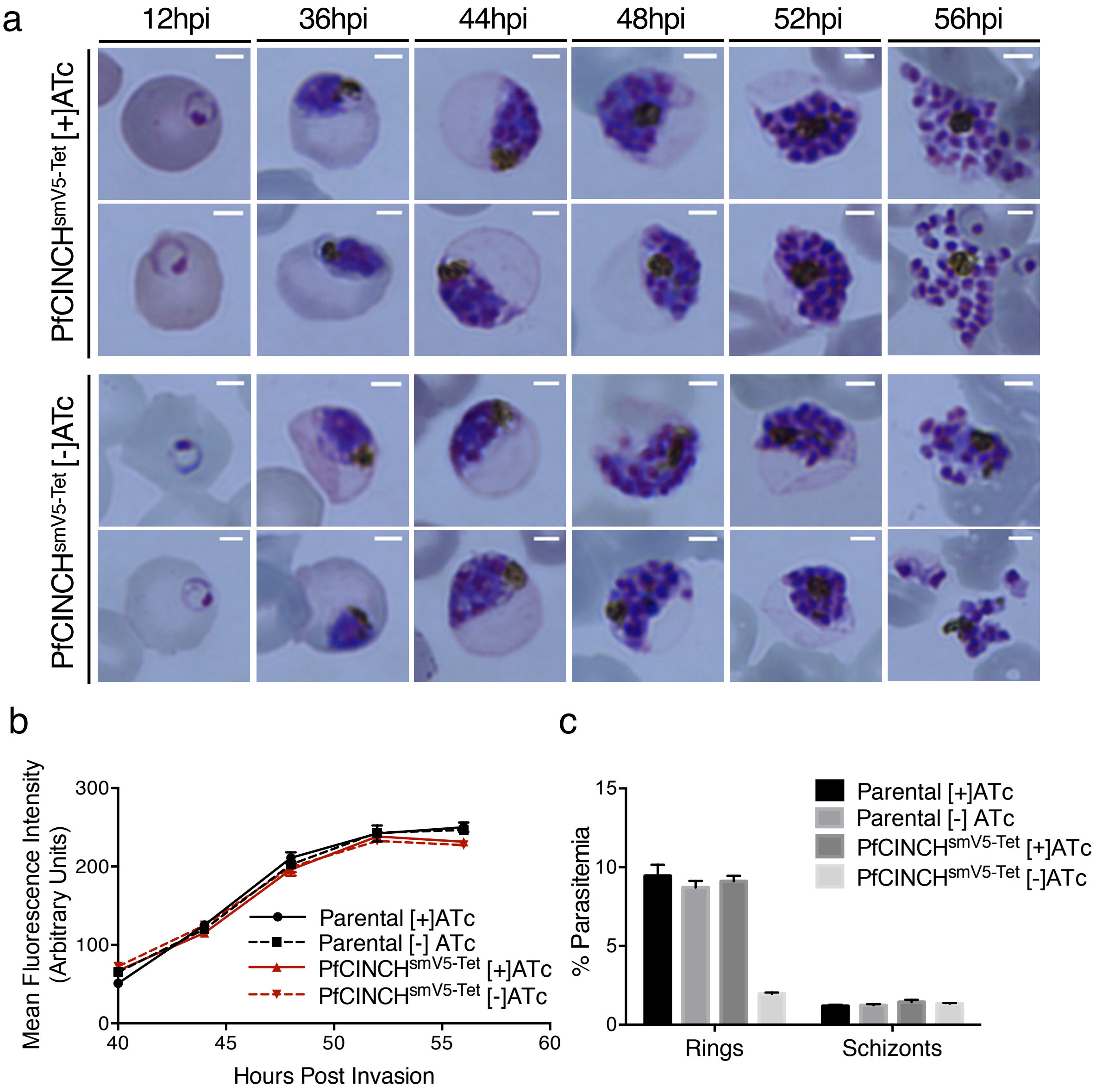

Supplementary Figure 4: PfCINCH-deficient parasites mature normally by Field's Stain and do not have abnormal nuclear content, but daughter cells fail to reinvade. a, Maturation of PfCINCHsmV5-Tet parasites [+]/[-]ATc throughout the asexual life cycle. Parasite growth slowed due to every 4 hour sampling. Scale bars 2μm. **b,** Mean fluorescence intensity of PfCINCH$^{smV5\text{-}Tet}$ and parental parasites [+]/[-]ATc throughout schizogony. No difference was observed in MFI between [+] and [-] conditions. Error bars show SD, n=3. **c,** Schizontemia and ring parasitemia at 56hpi following ATc washout. Error bars show SD, n=3. Source data are provided in the Source Data file.

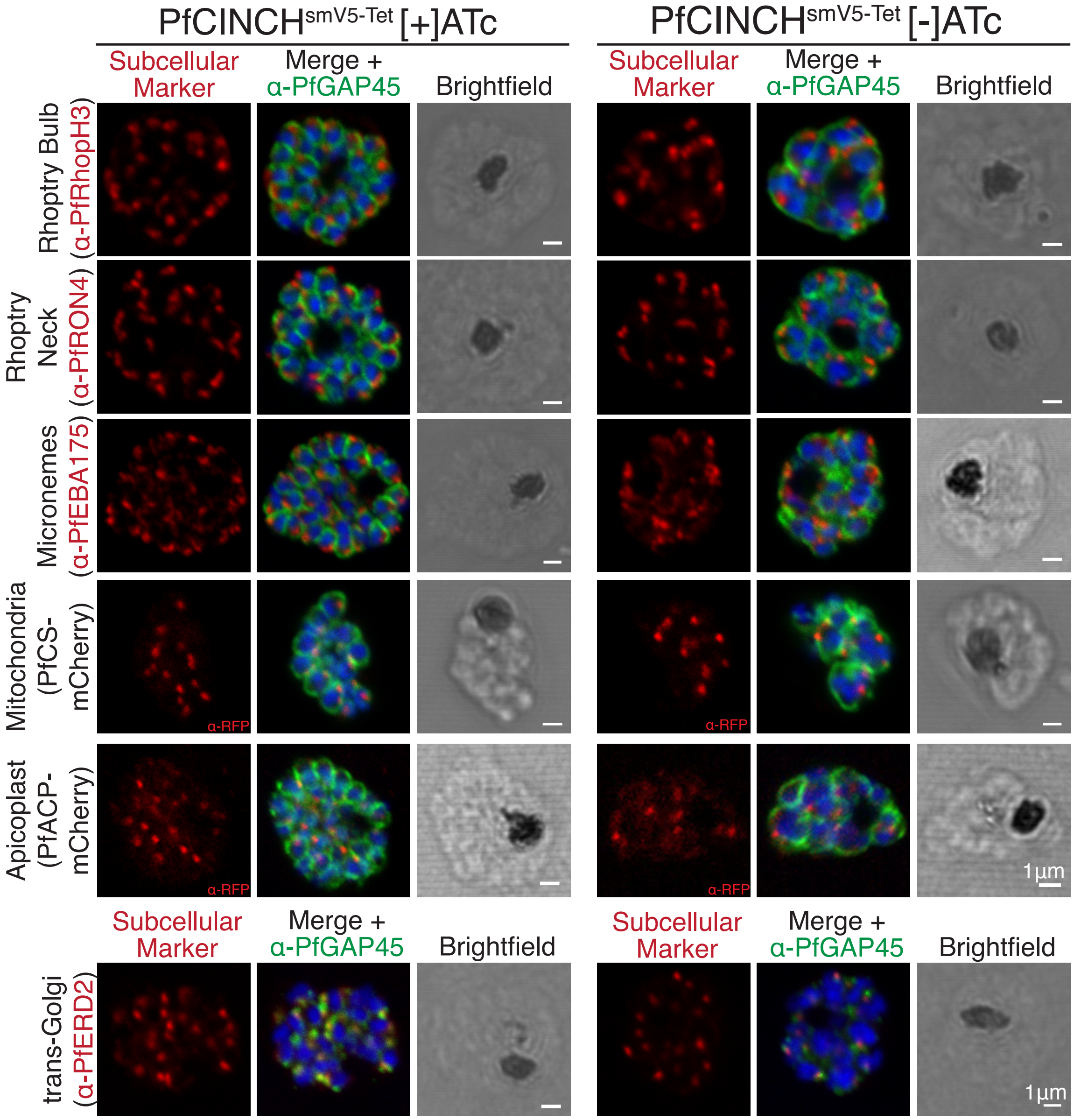

Supplementary Figure 5: Localization of subcellular markers in PfCINCH$^{smV5\text{-}Tet}$ parasites [+] and [-]ATc. Confocal microscopy of the rhoptry bulb (probed with α-PfRhopH3), rhoptry neck (α-PfRON3), micronemes (α-PfEBA175), mitochondria (parasites with episomal expression of PfCS-mCherry probed with a-RFP), apicoplast (parasites with episomal expression of PfACP-mCherry probed with α-RFP), and trans-Golgi (α-PfERD2). Images are of schizonts at the end of segmentation, determined by PfGAP45 surrounding merozoites or PfAMA1 staining. All scale bars 1μm.

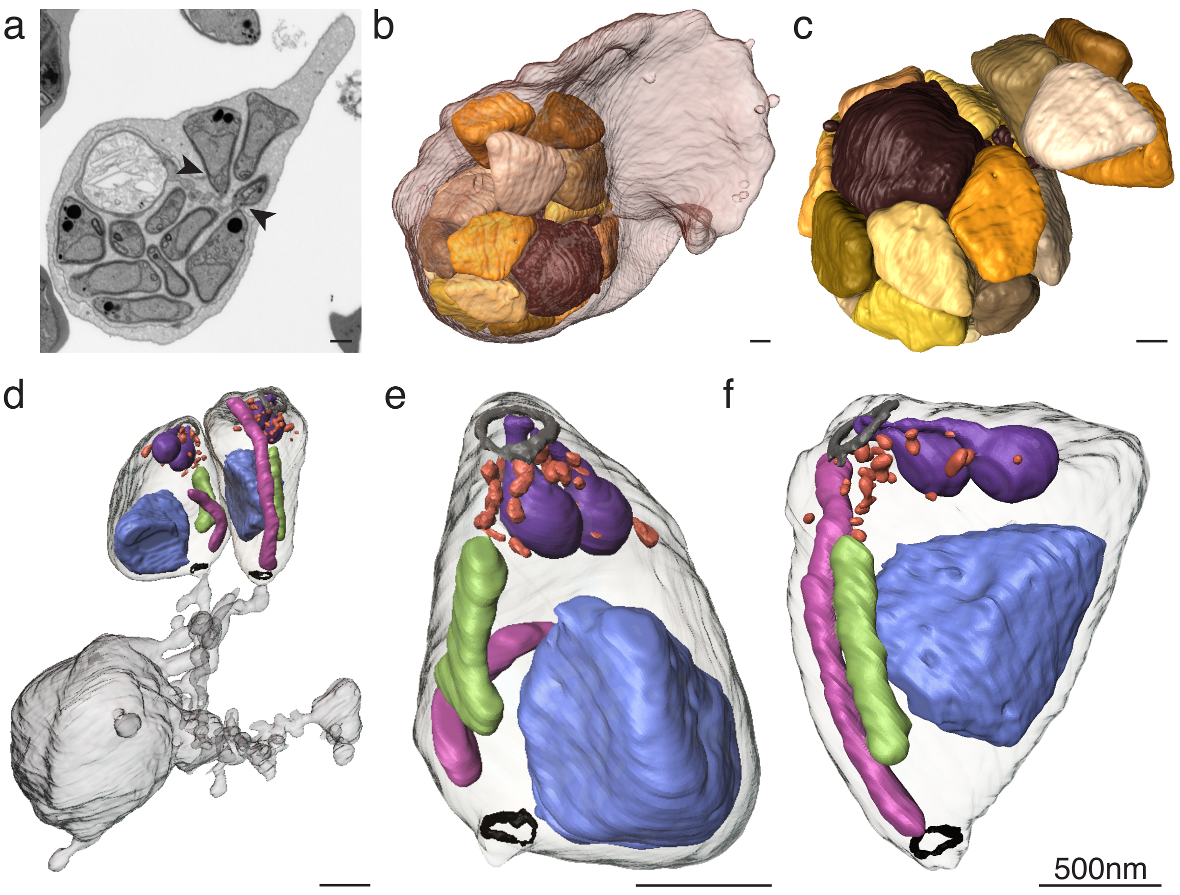

Supplementary Figure 6: 3D Ultrastructure of a [+]PfCINCH schizont. **a,** Representative EM slice with PVM mid-rupture (arrows). **b,** Rendered merozoites (shades of tan and brown) within red blood cell (transparent red). **c,** Rendered merozoites with food vacuole (dark brown) connecting to individual merozoites. **d,** Rendered food vacuole and connecting membranes (transparent) connected to merozoites. Two out of twenty four merozoites shown. **e,f,** Rendered single merozoite with apical ring (grey), basal ring (black), rhoptries (purple), small apical organelles (red), nucleus (blue), mitochondrion (pink), and apicoplast (green). Scale bars in renderings interpolated from structure sizes in SEM micrographs.

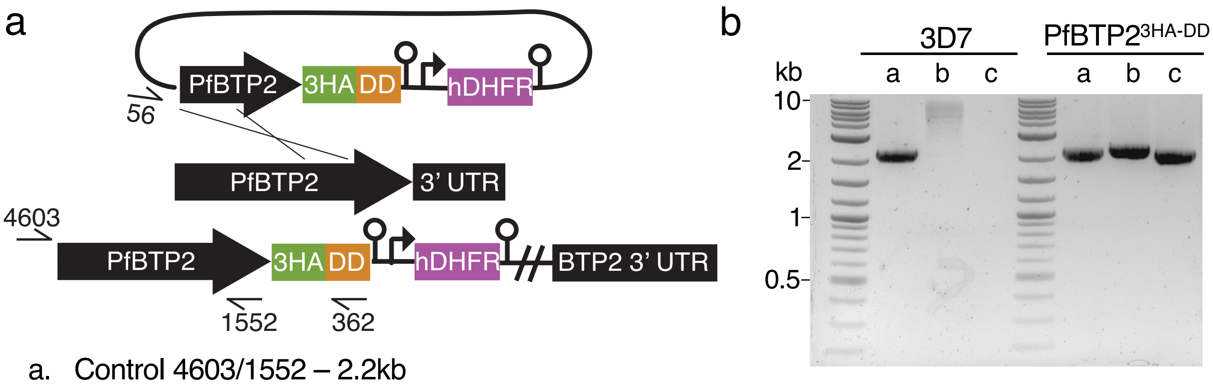

a. Control 4603/1552 – 2.2kb
b. Integration check 4603/362 – 2.4kb
c. Plasmid 56/1552 – 2.2kb

Supplementary Figure 7: Single crossover epitope-tagging of PfBTP2. a, Schematic of PfBTP2 single-crossover integration and expected sizes of PCR products. **b,** Agarose gel of PCR products to confirm plasmid integration into the PfBTP2 locus for parental (3D7) and transgenic (PfBTP2$^{3HA\text{-}DD}$) parasite lines. Source data are provided in the Source Data file.
