# Supplementary material for: An essential contractile ring protein controls cell division in Plasmodium falciparum
Source: Nat Commun. 2019 May 16;10:2181. doi: 10.1038/s41467-019-10214-z (PMC6522492; doi:10.1038/s41467-019-10214-z)
Supplement: Supplementary file 2 — Reporting Summary [file 41467_2019_10214_MOESM2_ESM.pdf]

nature research

Corresponding author(s): Jeffrey D. Dvorin

Last updated by author(s): Jan 2, 2019

Reporting Summary

Nature Research wishes to improve the reproducibility of the work that we publish. This form provides structure for consistency and transparency in reporting. For further information on Nature Research policies, see Authors & Referees and the Editorial Policy Checklist .

Statistics

For all statistical analyses, confirm that the following items are present in the figure legend, table legend, main text, or Methods section.

n/a	Confirmed	
☐	☒	The exact sample size (*n*) for each experimental group/condition, given as a discrete number and unit of measurement
☐	☒	A statement on whether measurements were taken from distinct samples or whether the same sample was measured repeatedly
☐	☒	The statistical test(s) used AND whether they are one- or two-sided<br>*Only common tests should be described solely by name; describe more complex techniques in the Methods section.*
☒	☐	A description of all covariates tested
☒	☐	A description of any assumptions or corrections, such as tests of normality and adjustment for multiple comparisons
☐	☒	A full description of the statistical parameters including central tendency (e.g. means) or other basic estimates (e.g. regression coefficient) AND variation (e.g. standard deviation) or associated estimates of uncertainty (e.g. confidence intervals)
☒	☐	For null hypothesis testing, the test statistic (e.g. *F*, *t*, *r*) with confidence intervals, effect sizes, degrees of freedom and *P* value noted<br>*Give P values as exact values whenever suitable.*
☒	☐	For Bayesian analysis, information on the choice of priors and Markov chain Monte Carlo settings
☒	☐	For hierarchical and complex designs, identification of the appropriate level for tests and full reporting of outcomes
☒	☐	Estimates of effect sizes (e.g. Cohen's *d*, Pearson's *r*), indicating how they were calculated

Our web collection on statistics for biologists contains articles on many of the points above.

Software and code

Policy information about availability of computer code

Data collection

Instrument: Software
Zeiss LSM800 with Airyscan: Zen black 2.3 V.14.0.9.201
Zeiss LSM700: Zen 2009 V. 6.0 SP2
LI-COR Odyssey CLx: Image Studio 4.0
Syngene G:Box: GeneSnap 7.12
Olympus BX40: INFINITY CAPTURE 6.5.4
BD FACS Calibur: CellQuest Pro 6.1
Nikon TiEclipse: NIS Elements v4.30
Zeiss Crossbeam 550: Atlas
FEI Helios Nanolab 660: Auto Slice & View 4
JEOL 1200EX: AMT Image Capture Engine v600.214
Zeiss AxioObserver: Zen 2.3 Pro

Data analysis

Data Type/ Instrument: Analysis Software
Immunofluorescence images from Zeiss LSM880 and Time-lapse microscopy from Zeiss AxioObserver: Zen Blue v2.3
Immunofluorescence images from Zeiss LSM700: Zen 2012 v2.3 lite and Adobe Photoshop CS6
Time-lapse microscopy from Nikon TiEclipse: NIS Elements v4.30
Field's stain images from Olympus BX40, transmission electron microscopy data from JEOL1200EX, and agarose gel images from Syngene G:Box : Adobe Photoshop CS6
Flow cytometry data from BD FACS Calibur: FlowJo X and GraphPad Prism 6
Immunoprecipitation data: Microsoft Excel V16.20 and GraphPad Prism 6
Quantitative Western blots from LICOR Odyssey CLx: Image Studio 4.0 and Adobe Photoshop CS6

FIB-SEM Data from Zeiss Crossbeam 550 and FEI Helios Nanolab 600: Image alignment, de-noising with non-local means filter, and binning performed with Matlab R2018a v9.4.0. Data segmentation and 3D rendering performed with FEI Avizo 9.1.1.

For manuscripts utilizing custom algorithms or software that are central to the research but not yet described in published literature, software must be made available to editors/reviewers. We strongly encourage code deposition in a community repository (e.g. GitHub). See the Nature Research guidelines for submitting code & software for further information.

Data

Policy information about availability of data

All manuscripts must include a data availability statement. This statement should provide the following information, where applicable:

- Accession codes, unique identifiers, or web links for publicly available datasets
- A list of figures that have associated raw data
- A description of any restrictions on data availability

All data are available upon request.

Field-specific reporting

Please select the one below that is the best fit for your research. If you are not sure, read the appropriate sections before making your selection.

[x] Life sciences [ ] Behavioural & social sciences [ ] Ecological, evolutionary & environmental sciences

For a reference copy of the document with all sections, see nature.com/documents/nr-reporting-summary-flat.pdf

Life sciences study design

All studies must disclose on these points even when the disclosure is negative.

Sample size

Description is for each figure.
Figure 1: representative example
Figure 2: Western blot (b) shown as representative sample. Representative replication curves (c) performed in technical triplicate for each condition. Developmental stage (d) represents 100 parasites for each time point. Smears were generated from technical triplicate from separate wells. Counting of egress events (f) completed for 25 events for each condition.
Figure 3: representative example
Figure 4: representative example
Figure 5: representative example of 2 cells that were segmented with Avizo software package.
Figure 6: Immunoprecipitation experiment performed twice. Data presented as mean +/- SD (b). Co-immunoprecipitation experiments performed in biological duplicate with representative immunoblot shown (c). Proteins present in both the sample and control data sets were excluded from b and c but are listed in Supplementary Excel File 1. Representative example (d and e).

Data exclusions

No data were excluded.

Replication

Description is for each figure.
Figure 1: representative example of immunofluorescence experiment performed >2 times.
Figure 2: Western blot replicated 3 times. Representative curves performed in technical triplicate for each condition (c and d). For egress counting events, 25 events for each condition were counted.
Figure 3: representative example of immunofluorescence experiment performed >2 times.
Figure 4: representative example of electron microscopy experiment performed on 3 samples per condition (one per time point).
Figure 5: representative example of FIB-SEM with >2 runs performed on same electron microscopy block
Figure 6: Immunoprecipitation experiments for sample and control repeated twice (b). Co-immunoprecipitation experiments performed in biological duplicate with representative immunoblot shown (c). Representative example of immunofluorescence experiment performed >2 times (d and e).

Randomization

The study did not involve randomization.

Blinding

The study did not involve blinded samples.

Reporting for specific materials, systems and methods

We require information from authors about some types of materials, experimental systems and methods used in many studies. Here, indicate whether each material, system or method listed is relevant to your study. If you are not sure if a list item applies to your research, read the appropriate section before selecting a response.

Materials & experimental systems

n/a	Involved in the study
☐	☒ Antibodies
☐	☒ Eukaryotic cell lines
☒	☐ Palaeontology
☒	☐ Animals and other organisms
☒	☐ Human research participants
☒	☐ Clinical data

Methods

n/a	Involved in the study
☒	☐ ChIP-seq
☐	☒ Flow cytometry
☒	☐ MRI-based neuroimaging

Antibodies

Antibodies used

Commercially available antibodies: Rat anti-HA (monoclonal 3F10, Roche/Sigma), Mouse anti-V5 (monoclonal SV5-PK1, Biorad), and rabbit anti-dsRed (recognizes mCherry, Clontech 632496). All secondary antibodies were purchased from Life Technologies.

Primary antibodies: Antibodies were generously provided by Robin Anders at The Walter & Eliza Hall Institute of Medical Research (mouse anti-PfAMA1 clone 1FG), Alan Cowman, Jenny Thompson and Kaye Wycherley at The Walter & Eliza Hall Institute of Medical Research (rabbit anti-PfEBA175, mouse anti-PfRON4), Julian Rayner at Wellcome Trust Sanger Institute (rabbit anti-PfGAP45), Anthony Holder at MRC National Institute for Medical Research (mouse anti-MSP1, clone 1E1), Odile Puijalon at Institut Pasteur Paris (mouse anti-PfRophH3) and Michael Makler at Flow Inc (mouse anti-PfLDH). Rabbit anti-ERD2 (MRA-1) was obtained through the Malaria Research and Reference Reagent Resource Center as part of the BEI resources, National Institute of Allergy and Infectious Diseases (NIAID), National Institutes of Health (NIH), contributed by John Adams.

Rabbit anti-PfMORN1 and Rabbit anti-PF3D7_1436200 antisera were raised for the current study.

Validation

Rabbit anti-PfMORN1 antiserum was validated by western blot against Plasmodium falciparum, recognizing the predicted band size at ~41 kD. The antiserum was further validated by observing the predicted localization pattern by immunofluorescence.

Rabbit anti-PF3D7_1436200 (BCP1) was validated by immunofluorescence assays showing the characteristic localization of basal complex proteins throughout the final stages of schizogony. The antiserum was further validated by the co-immunoprecipitation of PfCINCH-containing complexes. All of these validations were performed with pre-immune serum to demonstrate specificity.

Eukaryotic cell lines

Policy information about cell lines

Cell line source(s)	Plasmodium falciparum 3D7
Authentication	The 3D7 strain of P. falciparum was obtained from the Walter and Eliza Hall Institute (Melbourne, Australia).
Mycoplasma contamination	Not tested
Commonly misidentified lines (See ICLAC register)	*Name any commonly misidentified cell lines used in the study and provide a rationale for their use.*

Flow Cytometry

Plots

Confirm that:

☐ The axis labels state the marker and fluorochrome used (e.g. CD4-FITC).

☐ The axis scales are clearly visible. Include numbers along axes only for bottom left plot of group (a 'group' is an analysis of identical markers).

☐ All plots are contour plots with outliers or pseudocolor plots.

☐ A numerical value for number of cells or percentage (with statistics) is provided.

Methodology

Sample preparation	Parasite samples were stained with SYBR Green I (Life Technologies, S7563) at 1:1000 for 20 minutes at room temperature.
Instrument	BD FACSCalibur
Software	FACSCalibur run with CellQuest, analysis performed with FloJo X.

Cell population abundance	The relevant cell population is parasite infected red blood cells, determined by cells that are positive for SYBR Green staining. Un-infected red blood cells do not contain DNA and do not stain with SYBR green. Parasites are cultured in purified RBCs in RPMI, preventing signal from other DNA-containing cells. The percent parasitemia (the relevant population) ranged from 0.2% to 20%
Gating strategy	FSC/SSC gates were set to exclude debris and applied to all samples in a given data set. This gate typically included above 95% of the total measured cell population. We gated on signal in the 488 channel to determine if a red blood cell contained a parasite or not. A positive event had a 488 signal between 10-10,000.

☐ Tick this box to confirm that a figure exemplifying the gating strategy is provided in the Supplementary Information.
