# Supplementary material for: An essential contractile ring protein controls cell division in Plasmodium falciparum
Source: Nat Commun. 2019 May 16;10:2181. doi: 10.1038/s41467-019-10214-z (PMC6522492; doi:10.1038/s41467-019-10214-z)
Supplement: Supplementary file 3 — Description of Additional Supplementary Files [file 41467_2019_10214_MOESM3_ESM.pdf]

**Description of Additional Supplementary Files**

File Name: Supplementary Data 1
Description: Full dataset for PfCINCH coimmunoprecipitation

File Name: Supplementary Data 2
Description: Oligonucleotides and synthesized genes

File Name: Supplementary Movie 1
Description: Egress of [+]ATc PfCINCH$^{smV5\text{-}Tet}$ parasites. Video microscopy to monitor egress of schizonts treated with C1 or after C1 washout

File Name: Supplementary Movie 2
Description: Egress of [-]ATc PfCINCH$^{smV5\text{-}Tet}$ parasites. Video microscopy to monitor egress of PfCINCH-deficient schizonts treated with C1 or after C1 washout

File Name: Supplementary Movie 3
Description: 3D-rendering of a PfCINCH-deficient schizont. Rendering generated from FIB-SEM data of a PfCINCH-deficient schizont. Merozoite membranes are shaded in tan, nuclei in blue, apicoplasts in green, mitochondria in pink, rhoptries in purple, food vacuole/residual body in brown, and the red blood cell membrane in red.

File Name: Supplementary Movie 4
Description: FIB-SEM serial slices of a [+]ATc PfCINCH$^{smV5\text{-}Tet}$ parasite. Aligned serial sections of an E64-treated [+]ATc PfCINCH$^{smV5\text{-}Tet}$ schizont.

File Name: Supplementary Movie 5
Description: FIB-SEM serial slices of a [-]ATc PfCINCH$^{smV5\text{-}Tet}$ parasite. Aligned serial sections of an E64-treated [-]ATc PfCINCH$^{smV5\text{-}Tet}$ schizont.
